# Risk Factors Associated With Facial Acne Scarring in Thai Patients With Acne: A Cross‐Sectional Study

**DOI:** 10.1111/jocd.16695

**Published:** 2024-11-27

**Authors:** Chadakan Yan, Phichayut Phinyo, Yuri Yogya, Mati Chuamanochan, Rungsima Wanitphakdeedecha

**Affiliations:** ^1^ Center for Clinical Epidemiology and Clinical Statistics, Faculty of Medicine Chiang Mai University Chiang Mai Thailand; ^2^ Department of Biomedical Informatics and Clinical Epidemiology (BioCE), Faculty of Medicine Chiang Mai University Chiang Mai Thailand; ^3^ Musculoskeletal Science and Translational Research (MSTR) Center Chiang Mai University Chiang Mai Thailand; ^4^ Department of Dermatology and Venereology Universitas Padjadjaran Bandung Indonesia; ^5^ Division of Dermatology, Faculty of Medicine, Department of Internal Medicine Chiang Mai University Chiang Mai Thailand; ^6^ Faculty of Medicine Siriraj Hospital, Department of Dermatology Mahidol University Bangkok Thailand

**Keywords:** acne, acne scar, acne scarring, acne vulgaris, risk factor

## Abstract

**Background:**

One of the worst long‐term effects of acne is scarring, which leads to significant physical, psychological, and economic burdens. Limited studies have specifically studied the risk factors for acne scarring. This study aims to explore risk factors associated with facial acne scarring in Thai patients with acne.

**Methods:**

Exploratory cross‐sectional risk factor research was conducted using an online questionnaire on Thai patients aged ≥ 18 years who were diagnosed with acne between September and December 2023. The primary objective was to identify significant determinants of acne scars in patients with acne, including sociodemographic factors, clinical factors of acne, lifestyle factors, dietary habits factors, and treatment factors. Univariable and multivariable logistic regression were used to identify significant risk determinants.

**Results:**

Of 225 patients with 61.33% prevalence of acne scarring, acne scarring was found to be independently associated with the following variables: moderate acne (mOR 3.51, 95% CI 1.31–9.40, *p* = 0.012) or severe‐to‐very severe acne (mOR 8.98, 95% CI 2.71–29.73, *p* < 0.001), sometimes squeezing and picking behaviors (mOR 2.69, 95% CI 2.71–29.73, *p* = 0.033), and postacne erythema (PAE) (mOR 4.46, 95% CI 1.96–10.14, *p* < 0.001).

**Conclusion:**

The risk factors associated with acne scarring in individuals include the severity of acne, squeezing and picking behaviors, and experiencing PAE. One of this study's essential findings confirms that PAE is a notable component that could contribute to the development of acne scars. Early treatment of individuals at risk is crucial to reduce scar formation.

## 1 Introduction

Acne, usually called acne vulgaris, remains the most often treated inflammatory dermatosis worldwide [1]. The pathogenesis involves a complex interaction between hormonally induced sebum production, aberrant keratinization of the pilosebaceous duct, and immunological response to *Cutibacterium acnes* around the pilosebaceous unit [2]. The condition commonly develops during puberty, affecting nearly all adolescent boys (95%–100%) and a majority of teenage girls (83%–85%). It continues to persist in approximately 12%–14% of individuals throughout adulthood [3, 4].

One of the most severe sequelae of acne is scarring [5], a long‐lasting consequence that impacts nearly 95% of patients. Among acne patients, 47% get scars [6], and 30% have severe scarring [7, 8]. Acne's persistent nature and resulting scars cause substantial physical, psychological, psychological, social, and economic consequences [1]. Scars are formed due to an atypical healing response triggered by epidermal inflammation, resulting in an imbalance between matrix breakdown and collagen biosynthesis [9]. The development of scars can be categorized into two main stages: excessive tissue formation, resulting in keloid or hypertrophic scars, and tissue loss or destruction, leading to atrophic scars [5]. There are two main types of acne scars: hypertrophic scars/keloids and atrophic scars. Atrophic scars are observed in approximately 80%–90% of patients, and they are typically numerous [10]. The ice pick variety constitutes 60%–70% of atrophic scars, the boxcar accounts for 20%–30%, and the rolling variety comprises 15%–25% [11].

Society perceives facial acne scars as undesirable [12]. Few comprehensive studies have precisely examined the risk variables associated with acne scarring and the extent of its severity. Recent research validated the correlation between acne duration and treatment onset and showed that early treatment for inflammatory acne would effectively reduce the risk of physical and psychological scars [7, 13]. According to Heng et al. [6], family history and dietary habits were significant risk factors for developing acne, as well as its severity and potential scarring. There was a scarcity of studies that have mainly investigated the risk factors associated with acne scarring. Furthermore, no studies have examined the risk factors for acne scars within the Thai community. This study aimed to investigate the risk factors linked to acne and facial scarring in Thai individuals affected by acne. The results of this study will assist in formulating future approaches to diminish facial acne scarring in Thai acne patients and encourage timely intervention for individuals with a high susceptibility to scarring.

## 2 Methods

Exploratory risk factor research was conducted using a cross‐sectional design and a self‐reported questionnaire. The study's primary objective was to identify significant determinants of acne scars in Thai patients who responded to the survey between September and December 2023 in a dermatology outpatient clinic in a university hospital in Bangkok, Thailand, using convenient sampling. The inclusion criteria included Thai patients aged 18 years or older who were diagnosed with acne by physicians at the study site. The exclusion criteria were acne patients who could not read and upload clinical photos in the online questionnaire form. This study followed the Helsinki Declaration of 1964. The sample size calculation was based on data from previous relevant studies [5, 6, 7]. To achieve 80% statistical power and a two‐sided alpha error of 0.05 for identifying significant risk factors (i.e., gender, family history, the severity of acne, squeezing, and picking habits), at least 162 acne patients (75/87 in case/control) were required.

We collected all self‐reported demographic data and risk factors associated with postacne scarring from the literature review, including sociodemographic factors (age at acne onset [5], sex [5, 6], BMI [7], and family history of acne [6, 9]), clinical factors of acne (duration of acne [9], severity of acne [9], postacne erythema (PAE) [8], and squeezing and picking behaviors [9]), lifestyle factors (smoking/household smoker [7] and alcohol consumption [7]), dietary habits factors (butter consumption [6, 7], milk consumption [7], and cereal consumption [7]) and treatment factors (previous oral acne treatments [5] and previous topical acne treatments [5]). Each patient's clinical data and risk factors were collected from a self‐administered online questionnaire (using Google Forms). Photographs of the acne scar, levels of acne severity, and PAE were provided for respondents as an example of qualitative validation [9, 10]. To ensure the diagnosis of acne scarring, the clinical photo of the patient was validated by a dermatologist at the study site or the investigator via uploaded clinical photo (Y.Y.).

All statistical analyses were performed using Stata 17 (StataCorp, College Station, TX, USA). The normality of continuous data was examined by visualizing histograms. Continuous data regularly distributed was represented using the mean and standard deviation. Frequency and percentage were employed to characterize categorical data. Univariable and multivariable logistic regression were used to identify significant risk determinants. Odds ratios (OR), including univariable odds ratio (uOR) and multivariable odds ratio (mOR), along with their corresponding 95% confidence intervals (CI) for the risk factors associated with postacne scarring, were reported. As the objective was to explore independent risk factors, not to explain the causality of each factor, all factors within the multivariable model were presented regardless of their statistical significance. A *p* value of less than 0.05 was considered statistically significant.

## 3 Results

Two hundred twenty‐nine acne patients agreed to participate and responded to the online questionnaire. Four patients who did not complete the questionnaire were excluded. A total of 225 acne patients (98.25%) with the overall prevalence of acne scarring in the study site is 61.33% (case 138 persons/control 87 persons).

All demographic data and risk factors associated with postacne scarring, including sociodemographic factors, acne manifestation factors, lifestyle factors, dietary habits factors, and treatment factors, are shown in Table 1. Based on the univariable analysis, age at acne onset (≤ 15 years), family history of acne, the severity of acne (moderate to very severe), squeezing and picking behaviors, PAE, and treatment factors (previous topical and oral treatment) were found to be a risk factor for acne scarring (Table 1).

**TABLE 1:** Clinical characteristics and univariable analysis of factors of individual factors associated with acne patients with acne scarring compared to those without acne scarring.

Clinical characteristics	Acne patients with acne scarring (*n* = 138)	Acne patients without acne scarring (*n* = 87)	uOR (95% CI)	*p*
	*n* (%) or mean ± SD	*n* (%) or mean ± SD		
Sociodemographic factors				
Age of acne onset				
≤ 15	113 (81.88)	55 (63.22)	2.63 (1.42–4.86)	**0.002**
> 15	25 (18.12)	32 (36.78)	1.00 (Reference)	—
Sex				
Female	88 (63.77)	61 (70.11)	1.00 (Reference)	—
Male	50 (36.23)	26 (29.89)	1.33 (0.75–2.37)	0.328
BMI (kg/m^2^)	22.21 ± 3.91	21.79 ± 3.56		
< 18.5	18 (13.78)	13 (14.94)	1.00 (Reference)	—
18.5–23.0	79 (57.25)	51 (58.62)	1.11 (0.50–2.47)	0.782
> 23.0	41 (29.71)	23 (26.44)	1.28 (0.53–3.09)	0.572
Family history of acne	116 (84.06)	51 (58.62)	3.72 (1.99–6.95)	**< 0.001**
Clinical factors of acne				
Duration of acne (year)				
< 10	26 (18.84)	18 (20.69)	1.00 (Reference)	—
≥ 10	112 (81.16)	69 (79.31)	1.12 (0.57–2.20)	0.734
Severity of acne				
Almost clear, mild	41 (29.71)	73 (83.91)	1.00 (Reference)	—
Moderate	42 (30.43)	9 (10.34)	8.31 (3.68–18.78)	**< 0.001**
Severe, very severe	55 (59.86)	5 (5.75)	19.59 (7.26–52.82)	**< 0.001**
Squeezing and picking behaviors				
Never, rarely	40 (28.99)	42 (48.28)	1.00 (Reference)	—
Sometimes	51 (36.96)	25 (28.74)	2.14 (1.12–4.08)	**0.021**
Frequently, all the time	47 (34.06)	20 (22.99)	2.47 (1.25–4.87)	**0.009**
Postacne erythema	114 (82.61)	27 (31.03)	10.56 (5.61–19.87)	**< 0.001**
Lifestyle factors				
Smoking/household smoker	23 (16.67)	13 (14.94)	1.14 (0.54–2.39)	0.731
Alcohol consumption	75 (54.35)	49 (56.32)	0.92 (0.53–1.58)	0.772
Dietary habits factors				
Butter consumption				
Never/occasionally	55 (39.86)	39 (44.83)	1.00 (Reference)	—
More than once/week	83 (60.14)	48 (55.17)	1.23 (0.71–2.11)	0.462
Milk consumption				
Never/occasionally	27 (19.57)	17 (19.54)	1.00 (Reference)	—
More than once/week	111 (80.43)	70 (80.46)	0.99 (0.51–1.96)	0.996
Cereal consumption				
Never/occasionally	76 (55.07)	38 (43.68)	1.00 (Reference)	—
More than once/week	62 (44.93)	49 (56.32)	0.63 (0.67–1.09)	0.097
Treatment factors				
Previous topical acne treatments	119 (86.23)	45 (51.72)	5.85 (3.08–11.10)	**< 0.001**
Previous oral acne treatments^a^	78 (56.52)	21 (24.14)	4.09 (2.25–7.41)	**< 0.001**

*Note:* Statistically significant p‐value associations are shown in bold characters.

Abbreviations: BMI, body mass index; SD, standard deviation; uOR, univariable odds ratio.

^a^ Oral acne treatments included: antibiotics, oral contraceptives, and isotretinoin.

In multivariable analysis, the severity of acne (moderate to severe/very severe), squeezing and picking behaviors (sometimes), and PAE were identified as independent risk factors for acne scarring in multivariable analysis (Table 2). Severe‐to‐very severe acne severity showed the highest OR with mOR 8.98 (95% CI 2.71–29.73, *p* < 0.001) followed by PAE (mOR 4.46, 95% CI 1.96–10.14, *p* < 0.001), moderate acne severity (mOR 3.51, 95% CI 1.31–9.40, *p* = 0.012), and sometimes squeezing and picking behaviors (mOR 2.69, 95% CI 1.08–6.68, *p* = 0.033) (Table 2). Age at acne onset, family history of acne, squeezing and picking behaviors (frequently to all the time), and previous oral treatment showed no statistical significance after adjusting with all variables.

**TABLE 2:** Multivariable analysis of individual factors associated with acne patients with acne scarring compared to acne patients without acne scarring.

Clinical characteristics	mOR (95% CI)	*p*
Sociodemographic factors		
Age of acne onset		
≤ 15	2.24 (0.97–5.15)	0.059
> 15	1.00 (Reference)	—
Sex		
Female	1.00 (Reference)	—
Male	1.41 (0.56–3.56)	0.467
BMI (kg/m^2^)		
< 18.5	1.00 (Reference)	—
18.5–23.0	1.52 (0.52–4.41)	0.446
> 23.0	1.57 (0.44–5.61)	0.486
Family history of acne	1.31 (0.56–3.07)	0.540
Clinical factors of acne		
Duration of acne (year)		
< 10	1.00 (Reference)	—
≥ 10	0.92 (0.34–2.47)	0.862
Severity of acne		
Almost clear, mild	1.00 (Reference)	—
Moderate	3.51 (1.31–9.40)	**0.012**
Severe, very severe	8.98 (2.71–29.73)	**< 0.001**
Squeezing and picking behaviors		
Never, rarely	1.00 (Reference)	—
Sometimes	2.69 (1.08–6.68)	**0.033**
Frequently, All the time	2.05 (0.77–5.43)	0.150
Postacne erythema	4.46 (1.96–10.14)	**< 0.001**
Lifestyle factors		
Smoking/household smoker	0.72 (0.24–2.20)	0.567
Alcohol consumption	0.78 (0.36–1.73)	0.549
Dietary habits factors		
Butter consumption		
Never/occasionally	1.00 (Reference)	—
More than once/week	1.10 (0.45–2.71)	0.832
Milk consumption		
Never/occasionally	1.00 (Reference)	—
More than once/week	0.50 (0.16–1.54)	0.227
Cereal consumption		
Never/occasionally	1.00 (Reference)	—
More than once/week	0.90 (0.38–2.12)	0.804
Treatment factors		
Previous topical acne treatments	2.16 (0.85–5.48)	0.106
Previous oral acne treatments	1.08 (0.46–2.56)	0.852

*Note:* Statistically significant *p*‐value associations are shown in bold characters.

Abbreviation: mOR, multivariable odds ratio.

## 4 Discussion

This study was an exploratory risk factor research using a cross‐sectional design to find the risk factors of acne scarring in Thai patients. The risk factors for acne scarring in acne patients were the severity of acne (moderate to severe/very severe), squeezing and picking behaviors (sometimes), and PAE. We discovered a prevalence of 61.33% for overall acne scars, comparable to the 53.6%–60.7% prevalence of acne scars in a Chinese Singaporean [6] and Chinese Malaysian [7], respectively.

The findings indicate that moderate‐to‐severe acne, behaviors such as squeezing and picking, and postacne erythema (PAE) are significant risk factors for acne scarring. Our results align with those of Tan J. et al. [9] and Liu L. et al. [11], who also identified acne severity and squeezing/picking behaviors as key contributors to scarring, with Liu et al. [11] reported ORs of 2.34 and 5.51 for moderate and severe acne, respectively. Severe acne often results in prolonged inflammation [11], leading to irreversible damage to sebaceous glands and increased scarring risk [12]. Recent studies corroborate that early treatment for inflammatory acne reduces the likelihood of scarring [13, 14]. PAE, characterized by persistent telangiectasia and erythematous lesions, results from the sustained release of inflammatory cytokines such as interleukin‐6 and TNF‐α [15]. Although PAE frequently diminishes over time, it can persist for months and contribute to scar formation [15]. Tan J. et al. [9] found a significant association between PAE and acne scarring, emphasizing the importance of early treatment. Our findings support the view that PAE is a significant risk factor for acne scarring, necessitating consideration in patient management.

No significant associations were found between acne scarring and age at onset, sex, BMI, or family history in multivariable analysis. Previous studies suggested that men are more prone to acne scars due to higher testosterone levels [16] and unique sebaceous gland structures [17]. Moreover, women were more prone than males to be self‐conscious of their appearance and to seek medical attention from their doctors for acne [11]. Our findings did not support a gender‐based difference in scarring risk which is in contrast with the results of a few studies [5, 6, 7, 11]. Certain indicators indicated that there may be a close relationship between genetic variables, such as highly heritable traits, and particular genetic susceptibility loci (such as selectin L and transforming growth factor beta 2) [18]. A family history of acne was a risk factor in univariable analysis but not in multivariable analysis contrary to some studies [5, 6].

Lifestyle factors such as smoking and alcohol consumption were not associated with acne scarring in our study, aligning with some previous research [5, 6] but conflicting with others, such as Say Y et al. [7], which found maternal smoking to be protective. The relationship between nutrition and acne remains debated, with some studies suggesting a link between dairy and high glycemic index diets and increased acne risk through the insulin‐like growth factor 1 (IGF1) signaling pathway [7], while others propose that omega‐3‐rich and low‐GI diets may offer protection [19]. Regarding treatment, our study did not find an association between acne scarring and the use of topical treatments like benzoyl peroxide, clindamycin, or topical isotretinoin. This contrasts with Dessinioti C. et al. [5], which found that oral acne treatments, such as antibiotics and isotretinoin, were associated with higher odds of scarring. Therefore, the influence of factors such as genetics, lifestyle, and treatment modalities requires further investigation.

In addition, we discovered a correlation with the severity of acne but not with its duration. The duration and severity of acne scarring may contribute to the affected individual's acne‐scar development [9]. Individuals who experience mild acne over an extended period may not develop acne scars, whereas those with extensive acne may develop them within a shorter timeframe. Further research is required to examine the correlation between the duration and severity of acne.

Our study has identified several risk factors for acne scarring in acne patients, including the severity of acne (ranging from moderate to extremely severe), the presence of squeezing and picking behaviors, and the potential role of PAE as critical factors that may contribute to the prevention of further acne aggravation and scar formation. Early acne treatment is strongly recommended for individuals with modifiable risk factors.

This study had some limitations. Firstly, the survey was self‐reported by the respondents. We overcame the incorrect diagnosis by self‐uploading the acne scar pictures on the face for dermatology validation. Secondly, recall bias can be found in previous acne history. Lastly, our study was conducted in a single study site, and mild severity of acne patients might have been missed in these cases from hospital visits, which can limit the generalizability of the results to the Thai population.

## 5 Conclusion

In conclusion, the risk factors associated with acne scarring in individuals include the severity of the acne, performing squeezing and picking behaviors, and experiencing PAE. One of this study's essential findings confirms that PAE is a notable component that could contribute to the development of acne scars. Our understanding of risk factors could aid in preventing or mitigating facial acne scarring and facilitate early treatment for those with a high susceptibility to scarring.

## Author Contributions

All authors contributed to the study's conception and design. C.Y., P.P., M.C., and R.W. performed material preparation, data collection, and analysis. P.P., M.C., and R.W. had full access to all the data in the study and took responsibility for the integrity of the data and the accuracy of the data analysis. C.Y. and Y.Y. wrote the first draft of the manuscript, and all authors commented on previous versions. All authors read and approved the final manuscript. Authorship: All authors meet the International Committee of Medical Journal Editors criteria for authorship for this article, take responsibility for the integrity of the work as a whole, and have given their approval for this version to be published.

## Ethics Statement

The study was approved by the ethics committee of the Siriraj Institutional Review Board (SI 734/2023). This study followed the Helsinki Declaration of 1964 and its subsequent amendments.

## Conflicts of Interest

The authors declare no conflicts of interest.

## Data Availability

The datasets generated and analyzed during the current study are available from the corresponding author upon reasonable request.
